# Chronic hepatitis B infection and liver cancer

**DOI:** 10.2349/biij.2.3.e7

**Published:** 2006-07-01

**Authors:** CH Wong, KL Goh

**Affiliations:** Department of Medicine, Faculty of Medicine, University of Malaya, Kuala Lumpur, Malaysia

**Keywords:** Chronic hepatitis B infection, hepatocellular carcinoma

## Abstract

Hepatitis B virus (HBV) is one of the most well recognised human carcinogens. Since its discovery about 40 years ago, HBV has been studied extensively. This article summarises the evidence derived from various studies including epidemiological, animal model, histopathology studies and molecular genetics studies leading to the establishment of HBV as the main aetiological agent for hepatocellular carcinoma (HCC). The reduction in the incidence of childhood HCC due to mass hepatitis B vaccination in Taiwan is a dramatic demonstration of the critical aetiological role of hepatitis B in HCC. Thus it is essential for interventionalists to understand the epidemiological and pathogenesis of HCC to ensure optimal patient care.

## INTRODUCTION

Hepatocellular Carcinoma (HCC) is one of the most common cancers affecting Asians. In the overwhelming majority of cases, it is associated with chronic hepatitis B infection [1,2], which appears to be the cause of 50% to 60% of HCC worldwide [3]. Other recognised causes of HCC are chronic hepatitis C infection, alcoholic liver disease and other chronic liver diseases, which can lead to liver cirrhosis. HCC is a late complication of chronic hepatitis B infection that usually occurs at the fourth and fifth decade of life especially when the patients are older or liver cirrhosis has developed. The aim of this review is to provide the available evidence demonstrating the causal link between chronic hepatitis B infection and HCC.

## EPIDEMIOLOGICAL OBSERVATIONS

### Ecological Comparison Studies

The earliest epidemiological studies have shown that areas with a high prevalence of hepatitis B infection were also areas with the highest incidence of HCC (Table 1) [4,5]. For example, countries in the East Asian region - China, Taiwan, Korea and Japan where the prevalence rate of hepatitis B infection is very high, were also areas with the highest incidence of HCC. The burden of HCC in this region in fact, constitutes about 66% of the total number of HCC cases worldwide [6]. The risk factor for the high incidence of HCC in this area is hepatitis B infection where the attributable risk from this viral infection ranged from 40% to 90% [7].

**Table 1:** Prevalence of hepatitis B and incidence of liver cancer.

**Country**	Prevalence of HBV (%)* [4]	**Liver cancer incidence ASR (per 100,000 population) [5]**	
		Male	Female
China	5.3-12	37.9	14.2
South Korea	2.6-5.1	47.1	11.4
Thailand	4.6-8	38.6	17.2
USA	0.2-0.5	5.5	2.0
UK	< 1	3.3	1.7
Germany	0.62	4.2	1.5
Canada	0.5-1.0	4.0	1.4

ASR – Age Standardised Rate

* - Estimate based on HbsAg prevalence

Another interesting observation which is closely related to the above observations is the preponderance of HCC amongst certain ethnic groups with a high prevalence of hepatitis B infection. This observation is well seen in the multiethnic countries of Southeast Asia of Malaysia and Singapore where three major Asian races have co-existed for more than two generations. In these countries, the Chinese have the highest incidence of HCC and the highest prevalence of hepatitis B infection compared with the Malays and Indians who have a much lower prevalence of hepatitis B infection [8].

### Longitudinal Cohort Follow-up Studies

Longitudinal cohort follow-up studies have provided the most persuasive evidence supporting the causal association between HBV infection and HCC. Beasley *et al* conducted one of the largest epidemiological studies so far, involving 22,707 Chinese male civil servants in Taiwan [9]. Three thousand four hundred and fifty four patients or 15.2% of the study population were HBsAg-positive. The initial results from this extensive study were published in 1981 after a mean duration of 3.3 years follow-up constituting 75,000 man-years follow-up. During this period of follow-up, there were 307 deaths, 41 due to primary HCC and 19 due to liver cirrhosis, which accounted for 19.5% of the total deaths. Of the 41 patients who died of HCC, 40 were HBsAg-positive and only one was HBsAg-negative. This gave rise to a highly significant calculated relative risk of 223 among patients who were HBsAg-positive and dying of primary HCC. Among the 19 patients who died of liver cirrhosis, 17 of them were HBsAg-positive. The HBsAg-positive status was therefore significantly associated with a marked increase in the incidence of death due to HCC and liver cirrhosis.

A further follow-up review in 1986 with approximately 202,000 man-years of follow-up at an average of 8.9 years per man showed a further 161 cases of HCC. One hundred and fifty two cases were HBsAg-positive, giving rise to an incidence of HCC of 494.5 per 100,000 population per year among HBsAg carriers compared with 5.3 among the non-HBsAg carriers. The relative risk of HCC among HBsAg-carriers compared to non-HBsAg carriers for this data was 98.4 (95% CI=50.2 to 193) [10].

This Taiwan prospective study provided the strongest evidence of the role of chronic HBV infection as an aetiological agent in the causation of HCC.

### Effect of mass vaccination on liver cancer incidence rates in children

With the identification of HBV infections as a major aetiological factor in HCC, a nationwide hepatitis B vaccination program was implemented in Taiwan, the first such program worldwide in1984. Within 10 years, this program had successfully reduced the HBV carrier rate in children from 10% to 1%. The implementation of such a comprehensive vaccination program in Taiwan has allowed important epidemiological observations to be made with respect to the incidence of HCC.

In Taiwan, the association of HBV and HCC is stronger in children than in adults. The rate of seropositivity for HBV nearly approached 100% in children with HCC as compared to 70% to 80% in adults. Although, the incidence of childhood HCC is low worldwide, the incidence of HCC in Taiwan is relatively high and therefore, any changes in the incidence rate would be easier to detect and measure.

**Figure 1:**
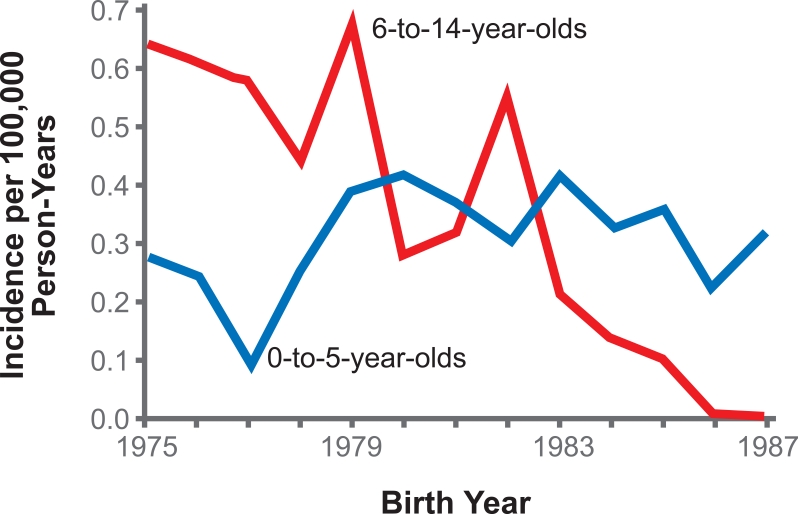
Comparison of the Incidence of Liver Cancer in Children 6 to 14 and 0 to 5 Years of Age, According to Birth Cohort [11] (reproduced with kind permission).

In 1994, Chang *et al* [11] studied the impact of universal hepatitis B vaccination in Taiwan on the incidence of HCC in children. In general, the incidence of HCC in children aged six to 14 years old showed a declining trend from 1981 to 1994. It was reported that the average annual incidence of HCC in these children between 1981 and 1986 was 0.70 per 100,000 children (range, 0.65 to 0.78). The incidence declined to 0.57 per 100,000 children (range, 0.48 to 0.62) between 1986 and 1990. This rate further dropped to 0.36 per 100,000 children (range, 0.23 to 0.48) between 1990 and 1994. This reduction in the annual incidence rate was highly significant statistically (p<0.01). The reduction in cancer incidence was seen with HCC only, while the annual incidence of other childhood cancers during this period remained similar indicating that the change in HCC incidence must be specifically related to the change in the prevalence of hepatitis B infection rather than a general change in the environment or living conditions. In addition, the reduction in the incidence of liver cancer was seen only in children aged six to 14 years old whereas the incidence remained unchanged in children aged up to five years old, in whom the aetiology of liver cancer was due to hepatoblastoma rather than hepatitis B infection (Figure 1).

## ANIMAL MODELS

The wood chuck hepatitis virus has similar characteristics with the human hepatitis B virus and provides an excellent animal model to study the pathogenesis of HCC. In the wild, animals infected with the woodchuck virus (WHV) have been shown to develop liver cancer. In experimental conditions, inoculation of a high dose of WHV results in the development of HCC in almost 100% of the animals. Laboratory studies have shown integration of the WHV DNA with the woodchuck hepatocyte DNA. The woodchuck model demonstrates to us that liver cancer can develop following a viral infection of the liver. [12, 13]

## HISTOPATHOLOGY STUDIES IN HUMANS

Scientists had postulated the association between HBV and HCC based on ecological comparison studies but were initially unable to demonstrate the virus within the HCC tissue until 1970s when a Japanese pathologist, Shikata, developed a histochemical stain called orcein stain [14], which could stain the HBsAg within the tissue and thereby allowing the identification of the virus in liver tissue. Subsequently, more sophisticated staining methods were developed such as immunoperoxidase technique and indirect immunofluorescence. These new techniques allowed scientists to study the association of HBV and HCC in greater depth. Many such studies have been published since then. One study done in Singapore [15] showed orcein-positive liver cells in 37 out of 50 (74.0%) cases of HCC, and only five out of 113 (4.4%) in the control group. In addition, there was also a significant difference in the frequency of HCC in orcein-positive and orcein-negative cirrhotic livers (28 out of 50 and 10 out of 40 respectively). Akagi *et al* [16] from Japan analysed 105 autopsy cases of HCC and found that HBsAg was detected with the orcein stain in 58 cases (55.2%) compared with four out of 171 cases (2.3%) of control group. These results demonstrated a close association between HBV and HCC.

## MOLECULAR STUDIES

Further evidence derived by using molecular studies has strengthened the causal relationship of HBV and HCC. It has been demonstrated that the incorporation of certain viral particle such as HBV DNA into the host genome can act as a precursor to HCC. The viral double stranded DNA encodes four types of genes i.e. surface gene for HBsAg, core gene for HBcAg, polymerase gene for HBV DNA and X gene for HBxAg. HBsAg, HBcAg, HBV DNA and HBxAg all have been found within HCC tumour in the tissue specimen. HBV DNA occurs mainly in two forms of infected hepatocytes, the covalently closed circular (ccc) DNA and the integrated DNA. The cccDNA serves as the template for host RNA polymerase II, which initiates the production of HBsAg, HBcAg, viral DNA polymerase and HBxAg. Whereas the integrated DNA predominates in HCC cell nuclei and has been shown in several studies to contain enhancers that may modulate several liver specific genes, transcriptional activators and alteration of the X gene functions. Although the actual mechanisms of hepatocarcinogenesis remain unclear, HBxAg have been implicated as the most important viral product that cause direct malignant cell transformation in the pathogenesis of HCC. Several in vitro studies have shown the effects of HBxAg on cellular repair, proto-oncogenes, tumour suppressor gene, anti-apoptotic pathway and the induction of HCC in transgenic mice. These evidence support the probable direct effect of a HBV component, HBxAg, in the development of HCC [17-24].

## EPILOGUE

The causal link between chronic hepatitis B infection and HCC is irrefutable. With mass vaccination at birth in place in most countries in Asia, and improved socio-economic conditions, the prevalence of hepatitis B infection will steadily decline. In countries where this has happened such as in Japan, hepatitis B is now the main “cause” of HCC as in the West. At present, however, hepatitis B remains prevalent in Asia and prevention will continue to result in marked decrease in the incidence of HCC.

## References

[1] Okuda K (1986). Primary liver cancer Quadrennial review lecture. Dig Dis Sci.

[2] Beasley RP, Hwang LY (1984). Hepatocellular carcinoma and hepatitis B virus. Semin Liver Dis.

[3] Hayashi PH, Di Bisceglie AM (2005). The progression of hepatitis B- and C-infections to chronic liver disease and hepatocellular carcinoma: epidemiology and pathogenesis. Med Clin North Am.

[4] World Health Organization Statistics by country or region WHO Statistical Information System 2004 [Web Page].

[5] International Agency for Research on Cancer (IARC). The GLOBOCAN 2002 database [Web Page].

[6] Bosch FX, Ribes J, Cleries R (2005). Epidemiology of hepatocellular carcinoma. Clin Liver Dis.

[7] Bosch FX, Ribes J, Borras J (1999). Epidemiology of primary liver cancer. Semin Liver Dis.

[8] Merican I, Guan R, Amarapuka D (2000). Chronic hepatitis B virus infection in Asian countries. J Gastroenterol Hepatol.

[9] Beasley RP, Hwang LY, Lin CC (1981). Hepatocellular carcinoma and hepatitis B virus A prospective study of 22 707 men in Taiwan. Lancet.

[10] Beasley RP (1988). Hepatitis B virus The major etiology of hepatocellular carcinoma. Cancer.

[11] Chang MH, Chen CJ, Lai MS (1997). Universal hepatitis B vaccination in Taiwan and the incidence of hepatocellular carcinoma in children Taiwan Childhood Hepatoma Study Group. N Engl J Med.

[12] Tennant BC, Toshkov IA, Peek SF (2004). Hepatocellular carcinoma in the woodchuck model of hepatitis B virus infection. Gastroenterology.

[13] Snyder RL, Tyler G, Summers J (1982). Chronic hepatitis and hepatocellular carcinoma associated with woodchuck hepatitis virus. Am J Pathol.

[14] Shikata T, Uzawa T, Yoshiwara N (1974). Staining methods of Australia antigen in paraffin section--detection of cytoplasmic inclusion bodies. Jpn J Exp Med.

[15] Tan AY, Law CH, Lee YS (1977). Hepatitis B antigen in the liver cells in cirrhosis and hepatocellular carcinoma. Pathology (Phila).

[16] Akagi G, Furuya K, Otsuka H (1982). Hepatitis B antigen in the liver in hepatocellular carcinoma in Shikoku, Japan. Cancer.

[17] Shafritz DA, Shouval D, Sherman HI (1981). Integration of hepatitis B virus DNA into the genome of liver cells in chronic liver disease and hepatocellular carcinoma Studies in percutaneous liver biopsies and post-mortem tissue specimens. N Engl J Med.

[18] Raimondo G, Burk RD, Lieberman HM (1988). Interrupted replication of hepatitis B virus in liver tissue of HBsAg carriers with hepatocellular carcinoma. Virology.

[19] Shamay M, Agami R, Shaul Y (2001). HBV integrants of hepatocellular carcinoma cell lines contain an active enhancer. Oncogene.

[20] Arbuthnot P, Capovilla A, Kew M (2000). Putative role of hepatitis B virus X protein in hepatocarcinogenesis: effects on apoptosis, DNA repair, mitogen-activated protein kinase and JAK/STAT pathways. J Gastroenterol Hepatol.

[21] Matsubara K, Tokino T (1990). Integration of hepatitis B virus DNA and its implications for hepatocarcinogenesis. Mol Biol Med.

[22] Gottlob K, Fulco M, Levrero M (1998). The hepatitis B virus HBx protein inhibits caspase 3 activity. J Biol Chem.

[23] Kim H, Lee H, Yun Y (1998). X-gene product of hepatitis B virus induces apoptosis in liver cells. J Biol Chem.

[24] Elmore LW, Hancock AR, Chang SF (1997). Hepatitis B virus X protein and p53 tumor suppressor interactions in the modulation of apoptosis. Proc Natl Acad Sci U S A.

